# Indoximod Attenuates Inflammatory Responses in Acetic Acid-Induced Acute Colitis by Modulating Toll-like Receptor 4 (TLR4) Signaling and Proinflammatory Cytokines in Rats

**DOI:** 10.3390/medicina61061033

**Published:** 2025-06-03

**Authors:** Gulcin Ercan, Hatice Aygun, Ahmet Akbaş, Osman Sezer Çınaroğlu, Oytun Erbas

**Affiliations:** 1 Department of General Surgery, Sultan 2. Abdulhamid Han Educational and Research Hospital, Istanbul Provincial Directorate of Health, Istanbul 34668, Turkey; ghepgul@hotmail.com; 2 Department of Physiology, Faculty Medicine, Tokat Gaziosmanpaşa University, Tokat 60250, Turkey; 3 Department of General Surgery, Faculty of Medicine, Karadeniz Technical University, Trabzon 61080, Turkey; draakbas@hotmail.com; 4 Department of Emergency Medicine, Faculty of Medicine, Izmir Katip Çelebi University, Izmir 35620, Turkey; drsezer@hotmail.com; 5 Faculty of Medicine, BAMER, Biruni University, Istanbul 34015, Turkey; oytunerbas2012@gmail.com

**Keywords:** Indoximod, TLR4, TNF-α, pentraxin-3, acetic acid-induced colitis, PAF, IDO inhibitor

## Abstract

*Background and Objectives:* Acute ulcerative colitis is characterized by excessive mucosal inflammation and epithelial disruption, often driven by dysregulated cytokine and immune signaling. Indoximod (1-methyl-DL-tryptophan), although not a direct enzymatic inhibitor, modulates the indoleamine 2,3-dioxygenase (IDO) pathway and has been reported to exert immunoregulatory effects in various models of inflammation. This study aimed to evaluate the protective effects of Indoximod in an acetic acid-induced colitis model in rats, focusing on histopathological changes and inflammatory mediators. *Materials and Methods:* Thirty male Wistar albino rats were randomly assigned to three groups (n = 10 per group): *Group 1 (Control)* received 0.9% saline oral gavage; *Group 2* *(Colitis)* received intrarectal 4% acetic acid to induce colitis and were then treated with saline; *Group 3 (Colitis + Indoximod)* received 4% acetic acid followed by oral gavage administration of Indoximod (30 mg/kg) for 15 consecutive days. Histopathological evaluation of colonic tissues was performed using hematoxylin and eosin (H&E) staining. Colonic expression of Toll-like receptor 4 (TLR4) and plasma levels of tumor necrosis factor-alpha (TNF-α), pentraxin-3 (PTX-3), and platelet-activating factor (PAF) were quantified using enzyme-linked immunosorbent assay (ELISA). *Results:* Acetic acid-induced colitis significantly increased mucosal damage, TLR4 expression, and circulating levels of TNF-α, PTX-3, and PAF compared with controls (*p* < 0.001). Indoximod treatment markedly reduced histological injury and significantly suppressed TLR4 and TNF-α levels (*p* < 0.01), along with partial reductions in PTX-3 (*p* < 0.05). However, PAF levels remained elevated despite treatment, indicating limited efficacy in PAF-associated pathways. *Conclusions:* Indoximod exhibited anti-inflammatory effects in this acute colitis model, likely by downregulating key proinflammatory mediators.

## 1. Introduction

Acute colitis refers to a sudden-onset inflammation of the colonic mucosa, which may arise in various clinical contexts, including infectious, ischemic, or inflammatory bowel diseases (IBD) such as ulcerative colitis. Ulcerative colitis, a major subtype of IBD, typically begins in the rectum and extends proximally throughout the colon [1]. According to the Global Burden of Disease Study, the prevalence of IBD has increased significantly in newly industrialized regions such as South America, Eastern Europe, Asia, and Africa [2]. Although current treatment options such as corticosteroids, 5-aminosalicylic acid derivatives, immunosuppressants, and anti-TNF-α agents can be effective, they are often associated with adverse effects and reduced efficacy over time [3,4]. This growing global burden underscores the urgent need for safer and more effective therapeutic strategies.

At the molecular level, proinflammatory cytokines, including tumor necrosis factor-alpha (TNF-α), interleukin-6 (IL-6), interleukin-1β (IL-1β), and interleukin-17 (IL-17) are key mediators in colitis pathogenesis. These cytokines promote inflammation by activating pattern recognition receptors such as Toll-like receptor 4 (TLR4), thereby amplifying the immune response and contributing to tissue injury in the colon [5]. Disruption of the intestinal epithelial barrier further exacerbates inflammation by facilitating microbial translocation and sustaining mucosal immune activation. In light of these mechanisms, increasing attention has been directed toward alternative therapies that offer both anti-inflammatory properties and improved safety profiles.

Indoximod (1-methyl-D-tryptophan; D-1-MT) is a synthetic tryptophan analog that modulates immune responses by functionally antagonizing indoleamine 2,3-dioxygenase 1 (IDO1), a rate-limiting enzyme in tryptophan catabolism [6]. The IDO1 pathway plays a pivotal role in maintaining mucosal immune homeostasis and epithelial integrity and has been identified as a relevant target in IBD models [7,8]. Elevated IDO1 expression and kynurenine metabolites have been observed in active colitis, highlighting its role in modulating T-cell responses and sustaining barrier function [7,9]. Experimental studies further suggest that manipulating this pathway can influence disease severity: IDO1 deficiency attenuates inflammation via downregulation of proinflammatory signaling [10], whereas its overexpression aggravates colitis through macrophage M1 polarization [7]. Moreover, studies have shown that acetic acid-induced colitis upregulates the IDO1/kynurenine axis, and agents like sodium selenite that inhibit this pathway effectively reduce mucosal inflammation and preserve epithelial integrity [9]. In line with this, Indoximod has demonstrated therapeutic benefits in DSS-induced colitis by enhancing mitochondrial bioenergetics and promoting mucosal healing [11]. Despite these promising findings, the therapeutic potential of IDO1 inhibitors such as Indoximod remains insufficiently explored in chemically induced models of acute colitis, particularly in acetic acid-induced colitis.

Therefore, this study aims to investigate the therapeutic efficacy of Indoximod in a rat model of acetic acid-induced acute colitis, with a focus on its impact on inflammatory cytokine profiles, epithelial damage, and mucosal immune regulation.

## 2. Materials and Methods

### 2.1. Animals

The study was conducted in accordance with the guidelines of the National Institutes of Health (NIH) for the care and use of laboratory animals and approved by the Institutional Animal Ethics Committee of Science University (Approval No: 2425025716; Approval Date: 18 March 2024). All experimental procedures were carried out in accordance with the ARRIVE guidelines. A total of thirty adult male Wistar albino rats (10–12 weeks old, weighing 150–200 g) were procured from the Experimental Animal Laboratory of Science University. The animals were housed in pairs within stainless steel cages in a temperature-controlled environment (22 ± 2 °C) under a standard 12-h light/dark cycle, with unrestricted access to food and water. Animals were allowed to acclimatize for one week prior to the experiment.

All animals were randomly assigned to experimental groups using a computer-generated randomization list. Allocation concealment was maintained by labeling cages with coded identifiers, which were unknown to the investigators conducting the outcome assessments. Both histological and biochemical analyses were performed in a blinded manner to ensure objectivity and minimize observational bias.

#### Safety and Tolerability

To monitor general health, body weights were recorded prior to each Indoximod administration. Humane endpoint criteria were predefined before the study and included (1) loss of more than 15% of initial body weight, (2) inability to walk or severe locomotor impairment, (3) significant reduction in food or water intake, and (4) decreased responsiveness to external stimuli. Animals meeting any of these criteria would be excluded from the study.

### 2.2. Drug

The selected dose of Indoximod (30 mg/kg/day) was based on previous toxicological studies in rodents. Jia et al. (2008) demonstrated that oral administration of D-1MT at doses ranging from 25 to 500 mg/kg/day for 28 consecutive days caused no mortality or observable toxicity in rats [12]. Furthermore, Ahlstedt et al. (2020) reported that daily intraperitoneal administration of approximately 30 mg/kg in rats for 10 days was well tolerated and did not induce inflammatory responses [13]. Based on these findings, a dose of 30 mg/kg/day was selected for the current study.

### 2.3. Induction of Experimental Colitis and Treatment Protocol

Experimental acute colitis was induced in 20 rats via intrarectal administration of 4% acetic acid (AA; Sigma-Aldrich, St. Louis, MO, USA). This model mimics the acute phase of ulcerative colitis by triggering localized mucosal injury, epithelial disruption, and an intense inflammatory response in the distal colon, thus serving as a reliable and reproducible method for studying the pathophysiology and potential therapeutic interventions of acute colonic inflammation [7,8]. Following light ether anesthesia, a soft polyethylene catheter was carefully inserted 6 cm into the rectum, and 1 mL of AA was administered slowly. To ensure homogeneous distribution of the solution and to prevent leakage, 1 mL of air was injected immediately afterward. The remaining ten animals served as the normal (non-colitic) control group (Group 1) and received 0.9% saline (1 mL/kg) by oral gavage once daily for 15 consecutive days [14,15].

After confirmation of colitis induction, the 20 colitis-induced rats were randomly allocated into two equal treatment groups (n = 10 per group) using a computer-generated randomization sequence:

Group 2 (Colitis group): Received 0.9% NaCl solution via oral gavage at a dose of 1 mL/kg/day.

Group 3 (Indoximod group): Received Indoximod via oral gavage at a dose of 30 mg/kg/day.

**Figure 1:**
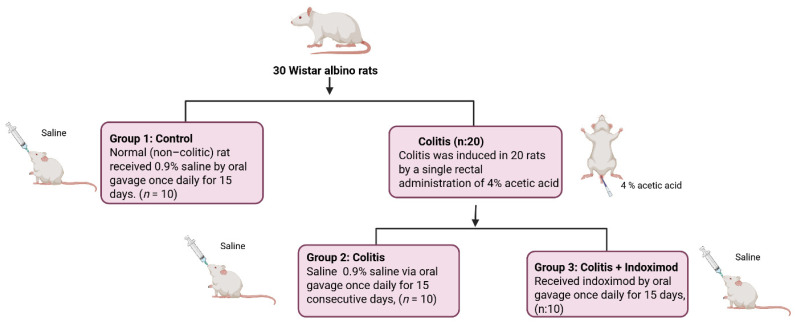
Schematic illustration of the experimental design. A total of 30 Wistar albino rats were assigned to three groups (n = 10 per group), with colitis induced in 20 rats via intrarectal administration of 4% acetic acid. Rats were then treated with either saline or Indoximod (30 mg/kg/day) by oral gavage for 15 days.

Treatments were administered via oral gavage for 15 consecutive days. At the end of the experimental period, all animals were euthanized under anesthesia. Blood was collected via cardiac puncture for biochemical analysis, and colonic tissues were excised for histopathological and biochemical evaluations (Figure 1).

### 2.4. Histological Examination of Colonic Tissue

For histological evaluation of mucosal integrity and inflammation, distal colon segments were carefully excised from each animal immediately after sacrifice. The samples were promptly rinsed with cold physiological saline to remove fecal material and blood residues, then immersed in 10% neutral-buffered formalin for 24 h at room temperature to ensure optimal tissue fixation. Formalin fixation preserves cellular architecture and prevents autolytic changes, allowing for accurate morphological assessment.

Following fixation, tissues were dehydrated through a graded series of ethanol, cleared in xylene, and embedded in paraffin blocks using standard histological processing protocols. Paraffin-embedded colon tissues were sectioned at a thickness of 4 µm using a rotary microtome to obtain thin, uniform slices suitable for microscopic analysis. Sections were subsequently mounted on glass slides and subjected to routine hematoxylin and eosin (H&E) staining. Hematoxylin stains nuclei dark blue, while eosin counterstains cytoplasmic and extracellular components pink, facilitating detailed visualization of inflammatory and structural changes [14,15].

Histological assessments were performed using an Olympus BX51 light microscope (Olympus Corporation, Tokyo, Japan) equipped with a digital camera system (Olympus C-5050), enabling both qualitative and quantitative evaluation of tissue architecture. To minimize bias, scoring was conducted blindly by an experienced pathologist who was unaware of the experimental groups. Colitis severity was evaluated according to the semi-quantitative grading system described by MacPherson and Pfeiffer (1976), which accounts for epithelial damage, leukocyte infiltration, and hemorrhage [15]:

0: Normal epithelium, no leukocyte infiltration or hemorrhage

1: <25% epithelial damage, focal leukocyte infiltration, and mild hemorrhage

2: ~25% epithelial loss, moderate focal infiltration, and hemorrhage

3: 50% epithelial loss with widespread leukocyte infiltration and hemorrhage

4: >50% epithelial destruction with extensive inflammatory cell infiltration and hemorrhage.

### 2.5. Quantification of TLR-4 in Colon Tissue

To evaluate innate immune activation, TLR-4 levels in colonic tissue were quantified using a rat-specific ELISA method. Frozen distal colon samples were homogenized in phosphate-buffered saline (PBS, pH 7.2) supplemented with protease inhibitors (1 mmol/L PMSF, 1 mg/L pepstatin A, aprotinin, and leupeptin) to prevent protein degradation. Homogenization was performed on ice using a glass Potter-Elvehjem homogenizer (Kimble Chase LLC, Rockwood, TN, USA) to preserve protein integrity. The homogenates were centrifuged at 12,000 rpm for 20 min at 4 °C, and the resulting supernatants were collected and either used immediately or stored at –80 °C.

Total protein concentrations were measured using the Bradford assay, with bovine serum albumin (BSA) as the standard. This normalization ensured that TLR-4 measurements reflected expression relative to total protein content. Quantification of TLR-4 was performed using a commercial ELISA kit (Thermo Fisher Scientific, Waltham, MA, USA), following the manufacturer’s protocol. All samples were analyzed in duplicate to ensure data reliability. Absorbance was recorded at 450 nm using a microplate reader, and concentrations were calculated from standard curves generated with recombinant rat TLR-4.

Final TLR-4 levels were expressed as picograms per milligram of tissue protein (pg/mg). These values enabled comparative analysis across experimental groups and provided insight into the modulation of colonic TLR-4 expression following experimental interventions.

### 2.6. Measurement of Plasma TNF-α, PTX3, and PAF

Plasma samples were obtained from blood collected via cardiac puncture in rats.

Plasma samples were collected in EDTA-containing tubes and centrifuged at 3000 rpm for 10 min at 4 °C. The supernatants were separated and stored at –80 °C until analysis. Plasma levels of tumor necrosis factor-alpha (TNF-α), Pentraxin-3 (PTX3), and Platelet-Activating Factor (PAF) were assessed using commercial ELISA kits (Biosciences, Charlestown, MA, USA) specific to rat proteins. All samples were assayed in duplicate according to the manufacturer’s protocols.

Following the final incubation step in the ELISA procedure, the optical density (OD) of each well was measured using a microplate reader (BioTek ELx808, Hampton, VA, USA) at a wavelength of 450 nm. Blank values were subtracted from sample absorbances to reduce background noise. Standard curves were generated for each analyte by plotting OD values against known concentrations of recombinant rat TNF-α, PTX3, and PAF standards provided with the kits. Linear regression analysis was used to derive equations from the standard curves, enabling the calculation of concentrations in experimental plasma samples.

Final results were expressed as picograms per milliliter (pg/mL) for TNF-α and PAF and nanograms per milliliter (ng/mL) for PTX3, based on their physiological abundance and standard calibration ranges. These values were then statistically analyzed to compare treatment effects across experimental groups.

### 2.7. Statistical Analysis

All statistical analyses were performed using both SPSS software (Version 19/USA) and GraphPad Prism (Version 10; GraphPad Software, San Diego, CA, USA). Data are expressed as mean ± standard error of the mean (SEM). The normality of data distribution within each group was assessed using the Kolmogorov–Smirnov test. Since all variables followed a normal distribution, parametric tests were applied. One-way analysis of variance (ANOVA) was used to compare differences among the three experimental groups (Control, Colitis, and Colitis + Indoximod), followed by Tukey’s post-hoc test for pairwise comparisons. A *p*-value < 0.05 was considered statistically significant. All graphical visualizations (box plots) were created using GraphPad Prism.

#### G*Power Analysis

Prior to initiating the study, an a priori sample size calculation was conducted using G*Power 3.1 software (3.1.9.7, Düsseldorf, Germany) based on a one-way ANOVA design. The parameters included a significance level of α = 0.05, a statistical power of 0.80, and a large effect size (f = 0.4), which was conservatively estimated based on previously published rat models of acetic acid-induced colitis [14,15,16]. These studies demonstrated statistically significant biochemical and histopathological outcomes with comparable sample sizes (typically 8–10 animals per group). Accordingly, the final sample size (n = 10 per group) was justified as adequate to detect biologically and statistically meaningful differences, consistent with the power analysis and prior experimental evidence.

To further validate sample adequacy, post hoc power analyses were performed using G*Power 3.1 for each primary outcome variable. Based on one-way ANOVA comparisons across the three groups (control, colitis, and colitis + Indoximod), all analyzed parameters, including histopathological scores, colon TLR-4 levels, and plasma concentrations of TNF-α, Pentraxin-3, and PAF—yielded large effect sizes (Cohen’s f ranging from 0.74 to 3.46) and high statistical power (1 − β ≥ 0.95). These results confirm the robustness and statistical validity of the findings obtained with the current sample size.

## 3. Result

### 3.1. Histopathological Score

**Figure 2:**
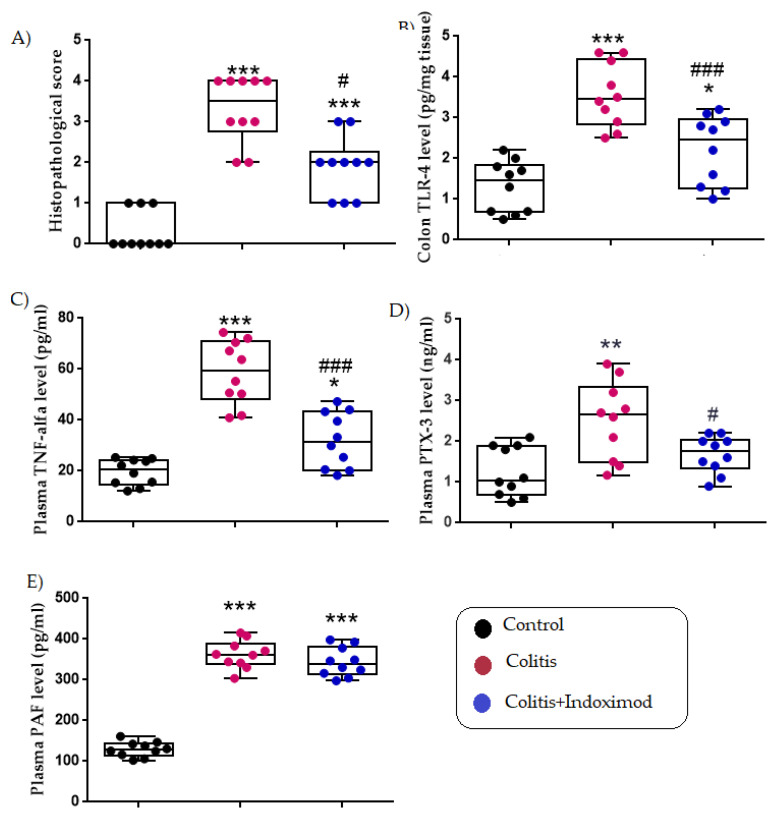
Effects of Indoximod on histopathological damage and inflammatory biomarkers in acetic acid-induced colitis. Box plots represent (**A**) histopathological score, (**B**) colonic TLR-4, (**C**) plasma TNF-α, (**D**) plasma PTX-3, and (**E**) plasma PAF levels. Data are expressed as mean ± SEM. Statistical analysis: one-way ANOVA with Tukey’s post hoc test. Significance indicators: *** *p* < 0.001, ** *p* < 0.01, * *p* < 0.05 different from control groups; ### *p* < 0.001, ## *p* < 0.01, # *p* < 0.05 different from colitis group.

**Figure 3:**
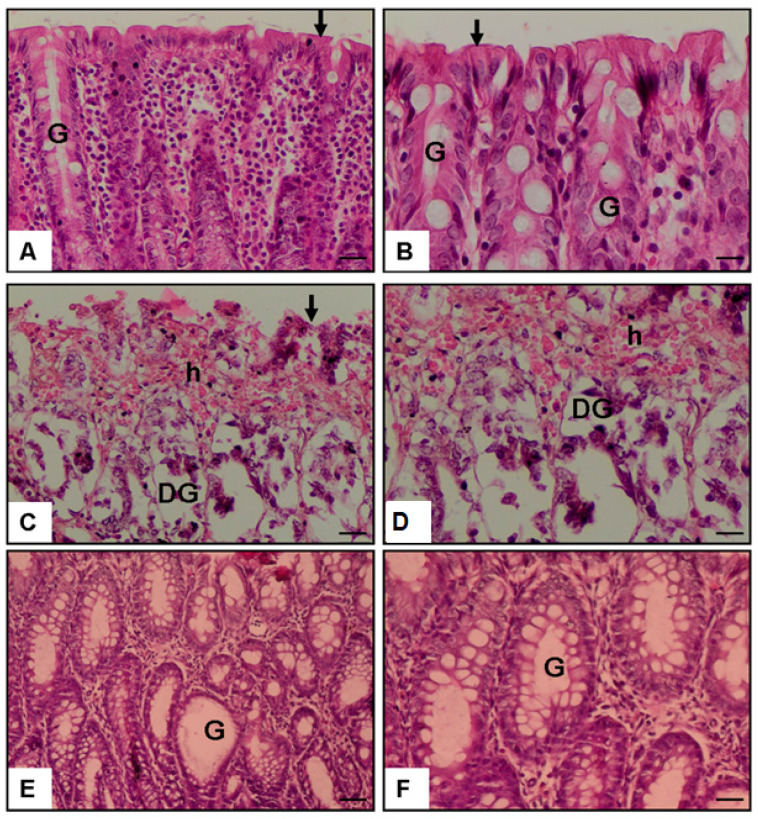
Representative H&E-stained colon sections at ×20 and ×40 magnification. (**A**,**B**) Control group: normal epithelial structure (arrow) and normal glands (G). (**C**,**D**) Colitis group: epithelial disruption (arrow), glandular degeneration (DG), and hemorrhage (h). (**E**,**F**) Colon sections from the colitis + Indoximod group display improved mucosal architecture with preserved epithelial lining and regenerated glands (G), indicating partial histological recovery. Scale bar = 50 µm.

The control group showed a mean histopathological score of 0.30 ± 0.15, reflecting preserved colonic architecture. In contrast, the colitis + saline group exhibited a significant increase to 3.30 ± 0.26 (*** *p* < 0.001; *p* = 0.0001), indicating severe mucosal injury. Indoximod treatment markedly reduced the score to 1.90 ± 0.23, with significant differences compared with both the colitis group (## *p* < 0.01; *p* = 0.005) and the control group (*** *p* < 0.001; *p* = 0.0001), supporting its protective efficacy (Figure 2 and Figure 3 and Table 1).

Figure 3 illustrates representative histological findings across the experimental groups. In the control group (Figure 3A,B), colonic sections show intact epithelial lining, well-preserved crypt architecture, and no signs of inflammation, indicating normal tissue morphology. In the colitis + saline group (Figure 3C,D), marked epithelial disruption, glandular degeneration (DG), and hemorrhage (h) are evident, along with dense leukocyte infiltration, reflecting severe mucosal injury. Sections from the Indoximod-treated group (Figure 3E,F) demonstrate partial mucosal recovery, with improved epithelial continuity and regenerating glands (G). Inflammatory infiltration appears reduced, supporting the histological improvement reflected in the scoring. These images, captured at ×20 and ×40 magnifications, visually confirm the protective effects of Indoximod on colonic histology.

### 3.2. Colon TLR-4 Level (pg/mg Tissue)

Colon TLR-4 levels were 1.31 ± 0.20 pg/mg in the control group and significantly elevated to 3.55 ± 0.25 pg/mg in the colitis + saline group (*** *p* < 0.001; *p* = 0.0001), reflecting a marked activation of innate immune signaling. This increase is consistent with the recognized role of TLR-4 in mediating mucosal inflammation. Indoximod treatment significantly decreased TLR-4 expression to 2.20 ± 0.27 pg/mg compared with the colitis group (## *p* < 0.01; *p* = 0.001), suggesting effective inhibition of TLR4-driven inflammatory responses. Interestingly, a mild but statistically significant reduction was also observed relative to the control group (* *p* < 0.05; *p* = 0.038), which may reflect an immunomodulatory baseline adjustment or a compensatory mechanism induced by Indoximod (Figure 2 and Table 1).

### 3.3. Plasma TNF-α Level (pg/mL)

Plasma TNF-α concentrations were 19.55 ± 1.63 pg/mL in the control group and significantly elevated to 58.69 ± 3.96 pg/mL in the colitis + saline group (*** *p* < 0.001; *p* = 0.0001), indicating pronounced systemic inflammation. Indoximod treatment substantially reduced TNF-α levels to 32.14 ± 3.45 pg/mL (### *p* < 0.001; *p* = 0.0001), showing a strong anti-cytokine effect. However, the levels remained higher than those of the control group (* *p* < 0.05; *p* = 0.024), suggesting that while inflammation was mitigated, complete cytokine normalization was not fully achieved. This partial reversal supports the immune modulatory effect of Indoximod (Figure 2 and Table 1).

### 3.4. Plasma Pentraxin-3 Level (ng/mL)

Plasma PTX-3 levels were 1.25 ± 0.19 ng/mL in the control group and increased to 2.51 ± 0.30 ng/mL in the colitis + saline group (** *p* < 0.01; *p* = 0.001), indicating an acute-phase inflammatory response. Treatment with Indoximod significantly reduced PTX-3 levels to 1.68 ± 0.14 ng/mL (# *p* < 0.05; *p* = 0.036), which reflects attenuation of the systemic inflammatory response. However, since PTX-3 levels did not return to baseline (*p* = 0.373 vs. control), this suggests a partial but incomplete resolution of inflammation, potentially due to persistent subclinical immune activation or tissue remodeling processes (Figure 2 and Table 1).

### 3.5. Plasma PAF Level (pg/mL)

PAF levels were 129.37 ± 5.83 pg/mL in control rats, rising significantly to 361.95 ± 10.85 pg/mL in the colitis + saline group (*** *p* < 0.001; *p* = 0.000), consistent with elevated PAF-mediated signaling in inflammatory settings. Indoximod treatment reduced PAF levels slightly to 343.63 ± 11.33 pg/mL, but this reduction was not statistically significant compared with the colitis group (*p* = 0.386), and levels remained significantly higher than the control (*** *p* < 0.001; *p* = 0.000). These results suggest that while Indoximod effectively modulates cytokine-related inflammation, its impact on lipid-derived mediators such as PAF may be limited, potentially pointing to pathway-specific selectivity (Figure 2 and Table 1).

### 3.6. General Health Observations and Tolerability

No mortality occurred in any experimental group throughout the study period. Daily monitoring revealed no weight loss exceeding 15% of baseline in any animal, and none met the predefined exclusion criteria. Furthermore, routine clinical observations did not identify any overt signs of toxicity, such as lethargy, impaired posture, or reduced grooming behavior. These findings complement the efficacy results and support the overall safety profile of Indoximod in this acute colitis model.

## 4. Discussion

This study evaluated the therapeutic potential of Indoximod in a rat model of acetic acid-induced acute colitis. As expected, the induction of acute colitis resulted in elevated levels of proinflammatory mediators such as TNF-α, PTX3, and PAF in plasma and TLR4 in colon tissue. Treatment with Indoximod significantly reduced these inflammatory markers and improved histopathological parameters, indicating a potent anti-inflammatory effect.

The acetic acid-induced colitis (AA-AC) model is a well-established in vivo system that recapitulates key features of acute human colitis, including epithelial disruption, mucosal ulceration, and prominent neutrophilic infiltration confined to the colonic mucosa [17]. Following chemical injury, rapid activation of the innate immune system triggers the recruitment of CD68⁺ macrophages and CD3⁺, CD4⁺, and CD8⁺ T lymphocytes to the site of inflammation [18]. Macrophages predominantly acquire a proinflammatory M1 phenotype, producing elevated levels of TNF-α, IL-1β, IL-6, IL-23, and reactive oxygen species (ROS), which intensify local tissue damage and promote further leukocyte infiltration [19,20]. Consequently, a broad spectrum of proinflammatory cytokines, including TNF-α, IL-1β, IL-6, IFN-γ, IL-17, IL-23, and the alarmin IL-33, is markedly upregulated, and neutrophil-mediated inflammation [19,20]. In contrast, regulatory cytokines such as IL-10 are significantly downregulated [21]. Histopathologically, the model is characterized by mucosal necrosis, collectively reflecting the innate-dominant inflammatory processes seen in the early stages of ulcerative colitis [22]. In our study, consistent with this profile, we observed significant increases in colonic proinflammatory mediator levels following AA administration. Hematoxylin and eosin staining confirmed pronounced epithelial injury, ulceration, and inflammatory infiltration in AA-AC rats. Indoximod treatment markedly reduced these pathological and molecular alterations, suggesting a protective effect likely mediated through anti-inflammatory and immunomodulatory mechanisms.

TLR4 is a key pattern recognition receptor that activates NF-κB signaling, leading to the release of proinflammatory mediators like TNF-α [23]. This pathway has been strongly implicated in colitis pathogenesis, particularly in the AA-AC model [24,25]. Pharmacological agents such as androstenediol, umbelliferone, and amitriptyline have been shown to attenuate colonic inflammation by modulating TLR4/NF-κB signaling [26,27,28]. In line with these studies, our data revealed increased colonic TLR4 and TNF-α levels in AA-AC rats, which were significantly reduced following Indoximod treatment. These findings suggest that Indoximod may exert its protective effects, at least in part, by downregulating the TLR4-driven inflammatory cascade.

Indoximod (D-1-methyltryptophan), although not a direct IDO1 inhibitor, modulates immune responses via alternative pathways, including suppression of TLR4/NF-κB signaling [6,29]. Prior studies have shown that both genetic and pharmacologic targeting of IDO1 reduced colonic inflammation by downregulating this pathway [10]. Wu et al. [30] further demonstrated that Indoximod decreased TNF-α levels and improved mucosal inflammation in the DSS-induced ulcerative colitis model. In our study, Indoximod treatment led to a marked decrease in both TLR4 and TNF-α expression in AA-AC rats, aligning with these findings.

PTX3, a member of the long pentraxin family, is rapidly induced by proinflammatory stimuli such as IL-1β, TNF-α, and TLR agonists [31,32]. Elevated PTX3 levels have been reported in acetic acid-induced colitis models, supporting its involvement in the acute inflammatory response [16]. Notably, PTX3 is known to amplify inflammation through a positive feedback loop involving the TLR4/NF-κB signaling axis [33]. In our study, Indoximod treatment markedly reduced PTX3 levels, suggesting that it may interrupt this feedback loop and thereby attenuate the overall inflammatory burden.

Although AhR and mTORC1 pathways were not directly assessed in our study, previous reports demonstrated that Indoximod activates AhR signaling and sustains mTORC1 activity, promoting IL-10–mediated anti-inflammatory responses and epithelial repair [29,34,35]. In parallel with these findings, we observed significant reductions in TNF-α and PTX3 levels, suggesting that the protective effects of Indoximod may involve indirect modulation of these immune-regulatory pathways.

Interestingly, while Indoximod significantly downregulated cytokine-driven inflammatory responses such as TNF-α, TLR4, and PTX3, it failed to modulate plasma PAF levels. This selective response likely reflects the mechanistic specificity of Indoximod, which primarily targets the IDO1–kynurenine axis—a pathway predominantly involved in adaptive immune regulation [7]. In the acetic acid-induced colitis model, inflammation is restricted to innate immune responses; T and B cell-mediated adaptive immunity is not a characteristic feature of this model [36,37]. Therefore, the limited efficacy of Indoximod on certain inflammatory mediators such as PAF may be attributable to the innate-dominant nature of the model, where adaptive immunomodulation exerts minimal influence.

Notably, PAF is a rapidly synthesized lipid mediator produced independently of canonical cytokine signaling, primarily through cytosolic phospholipase A₂ and acetyltransferase activation in response to acute membrane injury and oxidative stress [38]. In AA-AC, PAF levels rise sharply due to epithelial disruption and increased availability of membrane phospholipid substrates [39]. Although cytokines such as IL-1β and TNF-α can amplify PAF production, they are not essential for its synthesis, suggesting that PAF biosynthesis may persist despite effective cytokine suppression [40,41,42]. Moreover, previous studies demonstrated that pharmacological blockade of PAF receptors reduces neutrophil infiltration without preventing epithelial ulceration, underscoring the partial and context-specific role of PAF in acute colitis pathology [39]. Taken together, the unchanged PAF levels observed in our study likely do not reflect a failure of Indoximod’s therapeutic action but rather emphasize its selective influence on cytokine-mediated pathways without affecting lipid-derived inflammatory mediators. These findings highlight the importance of understanding mechanistic complementarity when evaluating anti-inflammatory efficacy in experimental models.

Importantly, throughout the 15-day treatment period, none of the animals exhibited mortality or clinical signs of toxicity, and all remained within the predefined humane endpoint criteria. These findings indicate a favorable safety profile of Indoximod at the administered dose. This outcome aligns with prior toxicological studies. Notably, Jia et al. (2008) demonstrated that oral administration of D-1-methyltryptophan (D-1MT) to rats at doses ranging from 25 to 500 mg/kg/day over 28 days produced no mortality, clinical toxicity, hematologic abnormalities, or histopathological alterations—establishing 500 mg/kg/day as the no-observed-adverse-effect level (NOAEL) [12]. Collectively, these data reinforce the safety and tolerability of Indoximod within the tested dose range.

## 5. Conclusions

Our findings demonstrate that Indoximod significantly reduced colonic TLR4 expression and plasma levels of TNF-α and PTX3 while also improving histopathological damage in rats with acetic acid-induced colitis. The unchanged PAF levels indicate that Indoximod’s effects may be limited to cytokine-mediated inflammation. These effects culminated in histological recovery and improved epithelial barrier integrity, suggesting that Indoximod holds therapeutic promise in the management of acute inflammatory colitis.

### Limitation

A key limitation of the present study is the exclusive use of male rats, which may limit the generalizability of the findings due to potential sex-based differences in immune responses and drug metabolism. Previous research has demonstrated that following dextran sodium sulfate (DSS)-induced colitis, female mice exhibited attenuated disease severity, including longer colon length, less body weight loss, lower stool consistency scores, reduced histopathological damage, and decreased colonic TNF-α levels compared with males. Notably, estradiol supplementation in ovariectomized females significantly ameliorated colitis severity [43]. Similarly, in a study using an acetic acid-induced colitis model, female rats showed higher hemoglobin and lymphocyte levels, as well as improved histological preservation, indicating a more favorable healing response than males [44]. These findings collectively underscore the importance of incorporating both sexes in future experimental colitis studies to enhance translational relevance and accurately capture sex-specific differences in disease progression and therapeutic response.

**Figure 4:**
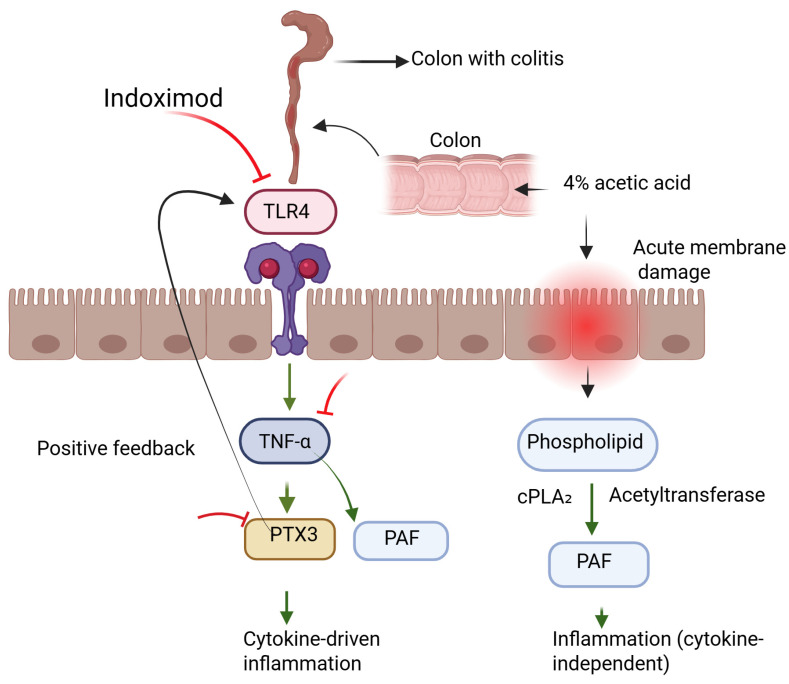
Graphical abstract image. Indoximod reduces TLR4-mediated inflammation in acetic acid-induced acute colitis.

Another important limitation concerns the acetic acid-induced colitis (AA-AC) model, which provides a convenient platform for studying acute mucosal injury and early inflammatory responses [37]. However, it has notable limitations in modeling chronic immune-mediated features of human inflammatory bowel diseases (IBD). The inflammation in this model is primarily driven by innate immune activation in response to chemical epithelial damage, lacking the T- and B-cell-mediated adaptive responses central to chronic IBD pathogenesis [36,37]. Moreover, the mucosal damage in AA-AC is typically transient and non-transmural, often resolving within two to three weeks [17], unlike the sustained and relapsing inflammation in ulcerative colitis or Crohn’s disease [20]. Consequently, while our findings highlight Indoximod’s efficacy in acute inflammation, extrapolation to human disease should be made cautiously. Future studies utilizing chronic or immune-driven models are needed to comprehensively evaluate its translational potential (Figure 4).

## Figures and Tables

**Table 1:** Histopathological scores and biochemical evaluations.

	Control Group	Colitis Group	Colitis + Indoximod Group
Histopathological score	0.30 ± 0.15	3.30 ± 0.26 ***	1.90 ± 0.23 ***,##
Colon TLR-4 level (pg/mg tissue)	1.31 ± 0.20	3.55 ± 0.25 ***	2.20 ± 0.27 *,##
Plasma TNF-alfa level (pg/mL)	19.55 ± 1.63	58.69 ± 3.96 ***	32.14 ± 3.45 *,###
Plasma Pentraxin-3 level (ng/mL)	1.25 ± 0.19	2.51 ± 0.30 **	1.68 ± 0.14 #
Plasma PAF level (pg/mL)	129.37 ± 5.83	361.95 ± 10.85 ***	343.63 ± 11.33 ***

Results were presented as mean ± SEM. Statistical analyses were performed by one-way ANOVA. ü Significance indicators: *** *p* < 0.001, ** *p* < 0.01, * *p* < 0.05 different from control groups; ### *p* < 0.001, ## *p* < 0.01, # *p* < 0.05 different from colitis group.

## Data Availability

Data available upon request from the authors.

## References

[1] Gajendran M., Loganathan P., Jimenez G., Catinella A.P., Ng N., Umapathy C., Ziade N., Hashash J.G. (2019). A comprehensive review and update on ulcerative colitis. Disease-a-Month.

[2] Alatab S., Sepanlou S.G., Ikuta K., Vahedi H., Bisignano C., Safiri S., Sadeghi A., Nixon M.R., Abdoli A., Abolhassani H. (2020). The global, regional, and national burden of inflammatory bowel disease in 195 countries and territories, 1990–2017: A systematic analysis for the Global Burden of Disease Study 2017. Lancet Gastroenterol. Hepatol..

[3] Xavier R.J., Podolsky D.K. (2007). Unravelling the pathogenesis of inflammatory bowel disease. Nature.

[4] Yukitake H., Kimura H., Suzuki H., Tajima Y., Sato Y., Imaeda T., Kajino M., Takizawa M. (2011). BTZO-15, an ARE-activator, ameliorates DSS- and TNBS-induced colitis in rats. PLoS ONE.

[5] Mukherjee T., Kumar N., Chawla M., Philpott D.J., Basak S. (2024). The NF-κB signaling system in the immunopathogenesis of inflammatory bowel disease. Sci. Signal..

[6] Fox E., Oliver T., Rowe M., Thomas S., Zakharia Y., Gilman P.B., Muller A.J., Prendergast G.C. (2018). Indoximod: An immunometabolic adjuvant that empowers T cell activity in cancer. Front. Oncol..

[7] Ciorba M.A. (2013). Indoleamine 2, 3 dioxygenase in intestinal disease. Curr. Opin. Gastroenterol..

[8] Gao Z., Shao S., Xu Z., Nie J., Li C., Du C. (2025). IDO1 induced macrophage M1 polarization via ER stress-associated GRP78-XBP1 pathway to promote ulcerative colitis progression. Front. Med..

[9] Ala M., Jafari R.M., Nematian H., Shadboorestan A., Dehpour A.R. (2022). Sodium selenite modulates IDO1/Kynurenine, TLR4, NF-κB and Bcl2/Bax pathway and mitigates acetic acid-induced colitis in rat. Cell Physiol. Biochem..

[10] Shon W.J., Lee Y.K., Shin J.H., Choi E.Y., Shin D.M. (2015). Severity of DSS-induced colitis is reduced in Ido1-deficient mice with down-regulation of TLR-MyD88-NF-kB transcriptional networks. Sci. Rep..

[11] Wu P., Yao S., Wang X., Yang L., Wang S., Dai W., Zhang H., He B., Wang X., Wang S. (2023). Oral administration of nanoformulated indoximod ameliorates ulcerative colitis by promoting mitochondrial function and mucosal healing. Int. J. Pharm..

[12] Jia L., Schweikart K., Tomaszewski J., Page J.G., Noker P.E., Buhrow S.A., Reid J.M., Ames M.M., Munn D.H. (2008). Toxicology and pharmacokinetics of 1-methyl-d-tryptophan: Absence of toxicity due to saturating absorption. Food Chem. Toxicol..

[13] Ahlstedt J., Konradsson E., Ceberg C., Redebrandt H.N. (2020). Increased effect of two-fraction radiotherapy in conjunction with IDO1 inhibition in experimental glioblastoma. PLoS ONE.

[14] Ercan G., Yigitturk G., Erbas O. (2020). Therapeutic effect of adenosine on experimentally induced acute ulcerative colitis model in rats. Acta Cir. Bras..

[15] MacPherson B., Pfeiffer C.J. (1976). Experimental colitis. Digestion.

[16] Ercan G., Aygün H., Akbaş A., Çınaroğlu O.S., Erbas O. (2025). Suramin Exerts an Ameliorative Effect on Acetic Acid-Induced Acute Colitis in Rats by Demonstrating Potent Antioxidant and Anti-Inflammatory Properties. Medicina.

[17] Cinpolat H.Y., Buğdaycı G., Şengül N., Astarcı H.M. (2023). A chemically induced experimental colitis model with a simple combination of acetic acid and trinitrobenzene sulphonic acid. Turk. J. Gastroenterol..

[18] Karaca T., Şimşek N., Uslu S., Kalkan Y., Can I., Kara A., Yörük M. (2012). The effect of royal jelly on CD3+, CD5+, CD45+ T-cell and CD68+ cell distribution in the colon of rats with acetic acid-induced colitis. Allergol. Immunopathol..

[19] Bertevello P.L., Logullo Â.F., Nonogaki S., Campos F.M., Chiferi V., Alves C.C., Torrinhas R.S., Gama-Rodrigues J.J., Waitzberg D.L. (2005). Immunohistochemical assessment of mucosal cytokine profile in acetic acid experimental colitis. Clinics.

[20] Randhawa P.K., Singh K., Singh N., Jaggi A.S. (2014). A review on chemical-induced inflammatory bowel disease models in rodents. Korean J. Physiol. Pharmacol..

[21] Colombo B.B., Fattori V., Guazelli C.F., Zaninelli T.H., Carvalho T.T., Ferraz C.R., Bussmann A.J.C., Ruiz-Miyazawa K.W., Baracat M.M., Casagrande R. (2018). Vinpocetine ameliorates acetic acid-induced colitis by inhibiting NF-κB activation in mice. Inflammation.

[22] Alsharif I.A., Fayed H.M., Abdel-Rahman R.F., Abd-Elsalam R.M., Ogaly H.A. (2022). Miconazole mitigates acetic acid-induced experimental colitis in rats: Insight into inflammation, oxidative stress and Keap1/Nrf-2 signaling crosstalk. Biology.

[23] Lin X., Kong J., Wu Q., Yang Y., Ji P. (2015). Effect of TLR4/MyD88 signaling pathway on expression of IL-1β and TNF-α in synovial fibroblasts from temporomandibular joint exposed to lipopolysaccharide. Mediat. Inflamm..

[24] Zhou P., Lai J., Li Y., Deng J., Zhao C., Huang Q., Yang F., Yang S., Wu Y., Tang X. (2022). Methyl gallate alleviates acute ulcerative colitis by modulating gut microbiota and inhibiting TLR4/NF-κB pathway. Int. J. Mol. Sci..

[25] Oubaid E.N., Abu-Raghif A., Al-Sudani I.M. (2023). Ibudilast ameliorates experimentally induced colitis in rats via down-reg-ulation of proinflammatory cytokines and myeloperoxidase enzyme activity. Pharmacia.

[26] Dejban P., Sahraei M., Chamanara M., Dehpour A., Rashidian A. (2021). Anti-inflammatory effect of amitriptyline in a rat model of acetic acid-induced colitis: The involvement of the TLR4/NF-kB signaling pathway. Fundam. Clin. Pharmacol..

[27] Abdel-Wahab B.A., Alkahtani S.A., Alqahtani A.A., Hassanein E.H. (2022). Umbelliferone ameliorates ulcerative colitis induced by acetic acid via modulation of TLR4/NF-κB-p65/iNOS and SIRT1/PPARγ signaling pathways in rats. Environ. Sci. Pollut. Res..

[28] Hassan H.A., Samy W., Mohammed H.O., Mahmoud S.M., Abbas N.A. (2024). Ameliorative effects of androstenediol against acetic acid-induced colitis in male wistar rats via inhibiting TLR4-mediated PI3K/Akt and NF-κB pathways through estrogen receptor β activation. Int. Immunopharmacol..

[29] Brincks E.L., Adams J., Wang L., Turner B., Marcinowicz A., Ke J., Essmann M., Mautino L.M., Van Allen C., Kumar S. (2020). Indoximod opposes the immunosuppressive effects mediated by IDO and TDO via modulation of AhR function and activation of mTORC1. Oncotarget.

[30] Wu W., Zhong W., Lin Z., Yan J. (2023). Blockade of Indoleamine 2, 3-Dioxygenase attenuates lipopolysaccharide-induced kidney injury by inhibiting TLR4/NF-κB signaling. Clin. Exp. Nephrol..

[31] Inoue K., Kodama T., Daida H. (2012). Pentraxin 3: A novel biomarker for inflammatory cardiovascular disease. Int. J. Vasc. Med..

[32] Doni A., Stravalaci M., Inforzato A., Magrini E., Mantovani A., Garlanda C., Bottazzi B. (2019). The long pentraxin PTX3 as a link between innate immunity, tissue remodeling, and cancer. Front. Immunol..

[33] Qi S., Zhao F., Li Z., Liang F., Yu S. (2020). Silencing of PTX3 alleviates LPS-induced inflammatory pain by regulating TLR4/NF-κB signaling pathway in mice. Biosci. Rep..

[34] Monteleone I., Rizzo A., Sarra M., Sica G., Sileri P., Biancone L., Macdonald T.T., Pallone F., Monteleone G. (2011). Aryl hydrocarbon receptor-induced signals up-regulate IL-22 production and inhibit inflammation in the gastrointestinal tract. Gastroenterology.

[35] Huynh L., Kusnadi A., Park S.H., Murata K., Park-Min K.H., Ivashkiv L.B. (2016). Opposing regulation of the late phase TNF response by mTORC1-IL-10 signaling and hypoxia in human macrophages. Sci. Rep..

[36] Elson C.O., Cong Y., McCracken V.J., Dimmitt R.A., Lorenz R.G., Weaver C.T. (2005). Experimental models of inflammatory bowel disease reveal innate, adaptive, and regulatory mechanisms of host dialogue with the microbiota. Immunol. Rev..

[37] Kawada M., Arihiro A., Mizoguchi E. (2007). Insights from advances in research of chemically induced experimental models of human inflammatory bowel disease. World J. Gastroenterol..

[38] Mascolo N., Izzo A.A., Autore G., Maiello F.M., Di Carlo G., Capasso F. (1995). Acetic acid-induced colitis in normal and essential fatty acid deficient rats. J. Pharmacol. Exp. Ther..

[39] Will P.C., Thomas T.K., Iverson L., Buckman D., Weis W., Wilson C., Srivastava A. (1991). Platelet activating factor as a proinflammatory mediator in acetic-induced colitis in the rat. Agents Actions.

[40] Lacasse C., Turcotte S., Gingras D., Stankova J., Rola-Pleszczynski M. (1997). Platelet-activating factor stimulates interleukin-6 production by human endothelial cells and synergizes with tumor necrosis factor for enhanced production of granulocyte-macrophage colony stimulating factor. Inflammation.

[41] Rehman I.U., Saleem M., Raza S.A., Bashir S., Muhammad T., Asghar S., Qamar M.U., Shah T.A., Bin Jardan Y.A., Mekonnen A.B. (2024). Anti-ulcerative colitis effects of chemically characterized extracts from C alliandra haematocephala in acetic acid-induced ulcerative colitis. Front. Chem..

[42] Vlachogianni I.C., Fragopoulou E., Stamatakis G.M., Kostakis I.K., Antonopoulou S. (2015). Platelet Activating Factor (PAF) biosynthesis is inhibited by phenolic compounds in U-937 cells under inflammatory conditions. Prostaglandins Other Lipid Mediat..

[43] Bábíčková J., Tóthová Ľ., Lengyelová E., Bartoňová A., Hodosy J., Gardlík R., Celec P. (2015). Sex differences in experimentally induced colitis in mice: A role for estrogens. Inflammation.

[44] Ige S., Aremu W., Olateju B., Oladipupo V.A., Adekola A.T. (2021). Effects of Age and Sex on the Healing of Acetic-Acid Induced Ulcerative Colitis in Adult Wistar Rats. Asian J. Med. Health.

